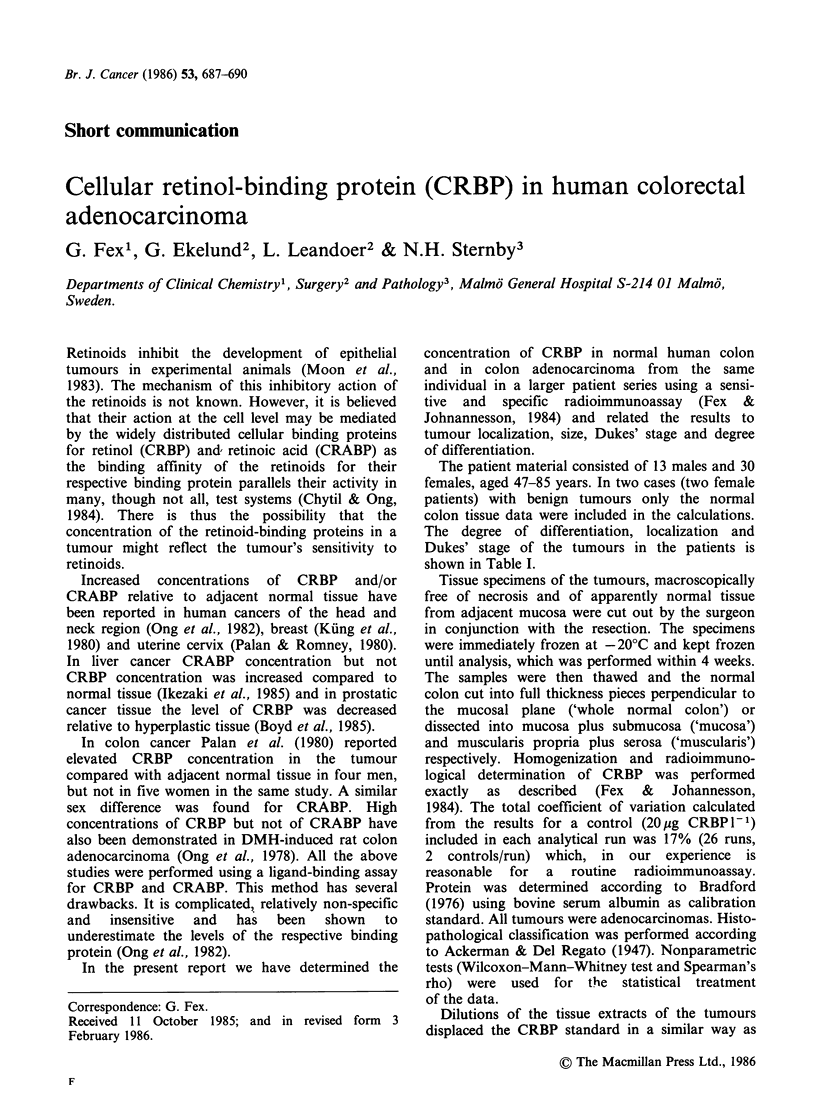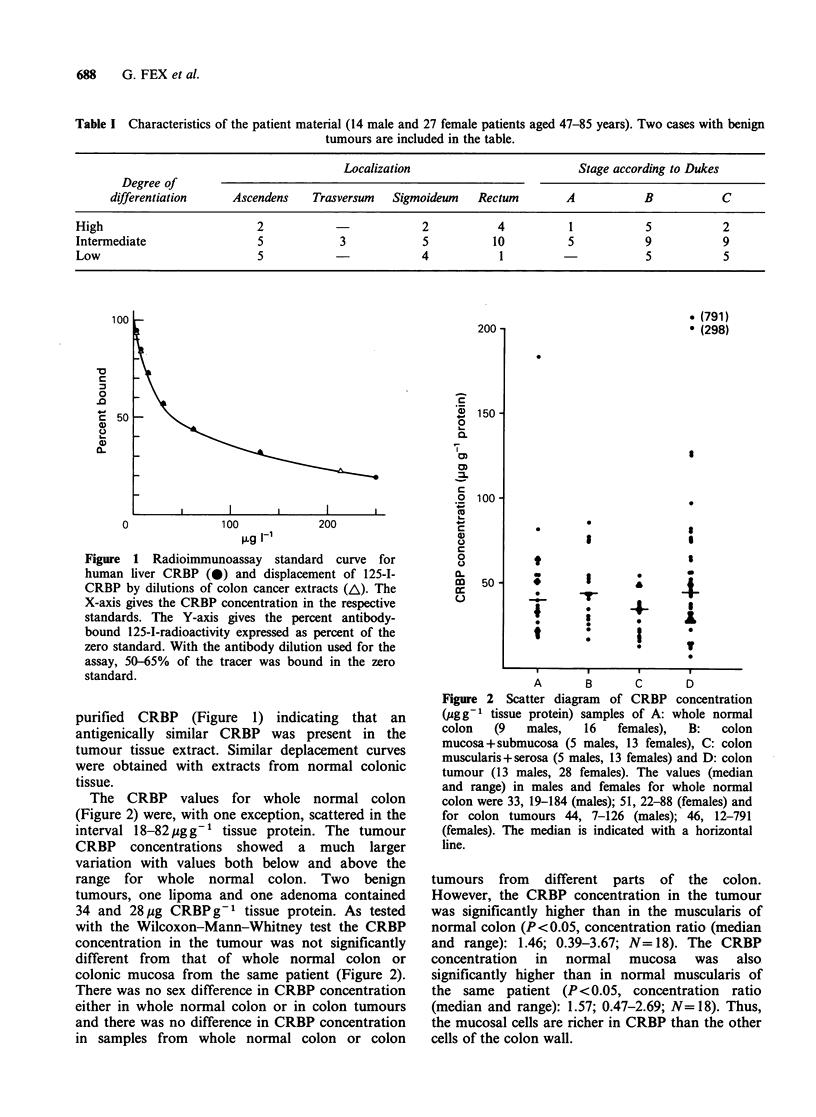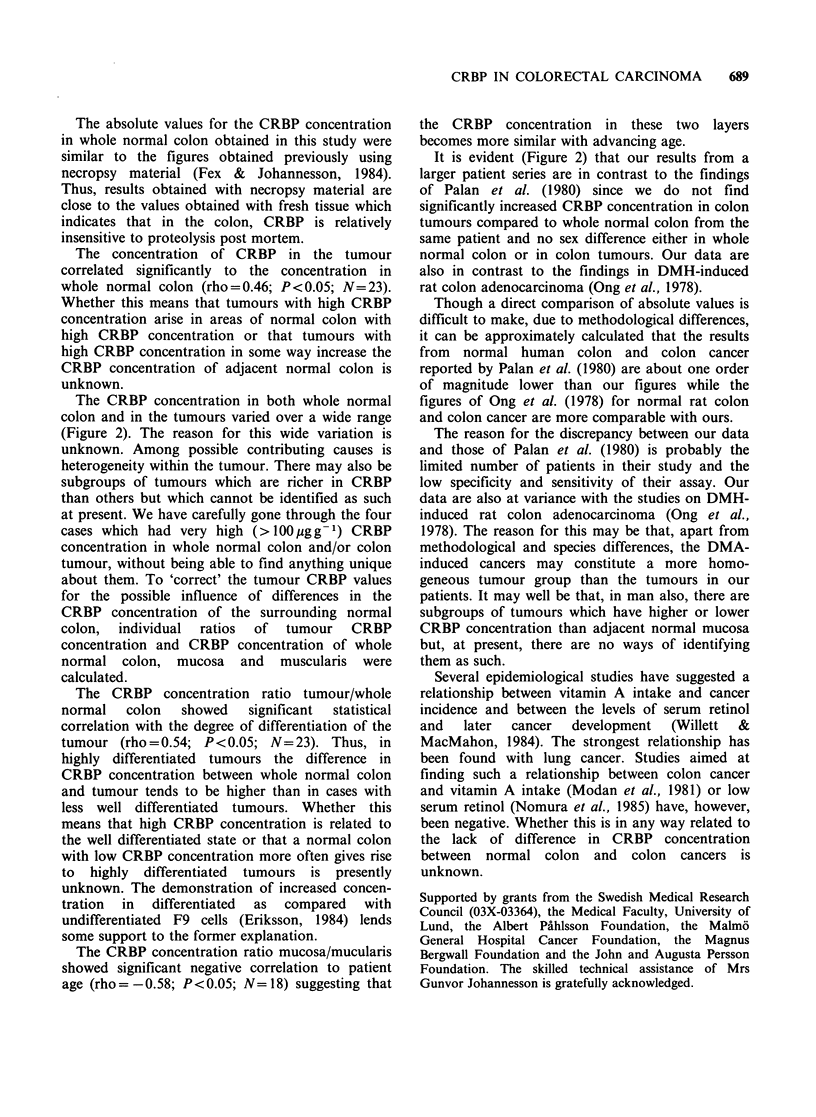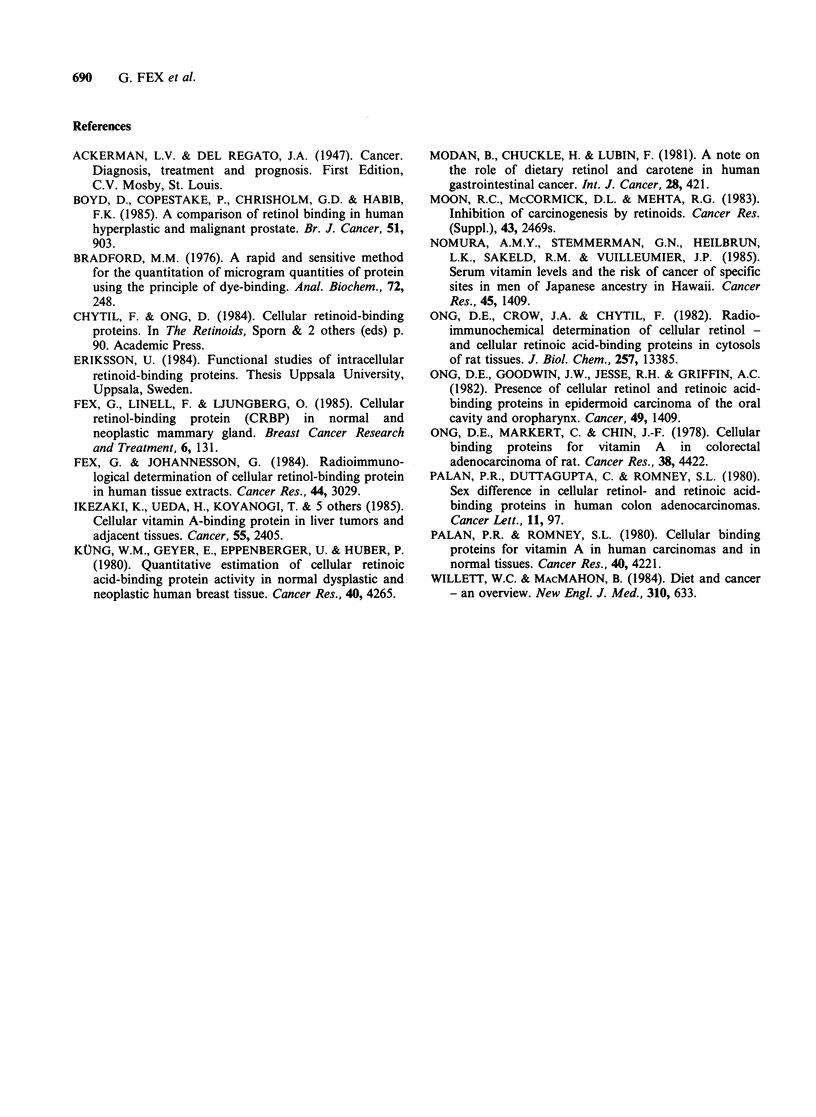# Cellular retinol-binding protein (CRBP) in human colorectal adenocarcinoma.

**DOI:** 10.1038/bjc.1986.113

**Published:** 1986-05

**Authors:** G. Fex, G. Ekelund, L. Leandoer, N. H. Sternby


*Br. J. Cancer* (1986) **53**, 687–690

**Short communication**

### Cellular retinol-binding protein (CRBP) in human colorectal adenocarcinoma

G. Fex[1], G. Ekelund[2], L. Leandoer[2] & N.H. Sternby[3]

*Departments of Clinical Chemistry[1], Surgery[2] and Pathology[3], Malmö General Hospital S-214 01 Malmö, Sweden.*

Retinoids inhibit the development of epithelial tumours in experimental animals (Moon *et al.*, 1983). The mechanism of this inhibitory action of the retinoids is not known. However, it is believed that their action at the cell level may be mediated by the widely distributed cellular binding proteins for retinol (CRBP) and retinoic acid (CRABP) as the binding affinity of the retinoids for their respective binding protein parallels their activity in many, though not all, test systems (Chytil & Ong, 1984). There is thus the possibility that the concentration of the retinoid-binding proteins in a tumour might reflect the tumour's sensitivity to retinoids.

Increased concentrations of CRBP and/or CRABP relative to adjacent normal tissue have been reported in human cancers of the head and neck region (Ong *et al.*, 1982), breast (Küng *et al.*, 1980) and uterine cervix (Palan & Romney, 1980). In liver cancer CRABP concentration but not CRBP concentration was increased compared to normal tissue (Ikezaki *et al.*, 1985) and in prostatic cancer tissue the level of CRBP was decreased relative to hyperplastic tissue (Boyd *et al.*, 1985).

In colon cancer Palan *et al.* (1980) reported elevated CRBP concentration in the tumour compared with adjacent normal tissue in four men, but not in five women in the same study. A similar sex difference was found for CRABP. High concentrations of CRBP but not of CRABP have also been demonstrated in DMH-induced rat colon adenocarcinoma (Ong *et al.*, 1978). All the above studies were performed using a ligand-binding assay for CRBP and CRABP. This method has several drawbacks. It is complicated, relatively non-specific and insensitive and has been shown to underestimate the levels of the respective binding protein (Ong *et al.*, 1982).

In the present report we have determined the concentration of CRBP in normal human colon and in colon adenocarcinoma from the same individual in a larger patient series using a sensitive and specific radioimmunoassay (Fex & Johnannesson, 1984) and related the results to tumour localization, size, Dukes' stage and degree of differentiation.

The patient material consisted of 13 males and 30 females, aged 47–85 years. In two cases (two female patients) with benign tumours only the normal colon tissue data were included in the calculations. The degree of differentiation, localization and Dukes' stage of the tumours in the patients is shown in Table I.

Tissue specimens of the tumours, macroscopically free of necrosis and of apparently normal tissue from adjacent mucosa were cut out by the surgeon in conjunction with the resection. The specimens were immediately frozen at $-20°C$ and kept frozen until analysis, which was performed within 4 weeks. The samples were then thawed and the normal colon cut into full thickness pieces perpendicular to the mucosal plane ('whole normal colon') or dissected into mucosa plus submucosa ('mucosa') and muscularis propria plus serosa ('muscularis') respectively. Homogenization and radioimmunological determination of CRBP was performed exactly as described (Fex & Johannesson, 1984). The total coefficient of variation calculated from the results for a control ($20\,\mu g$ CRBP $l^{-1}$) included in each analytical run was 17% (26 runs, 2 controls/run) which, in our experience is reasonable for a routine radioimmunoassay. Protein was determined according to Bradford (1976) using bovine serum albumin as calibration standard. All tumours were adenocarcinomas. Histopathological classification was performed according to Ackerman & Del Regato (1947). Nonparametric tests (Wilcoxon–Mann–Whitney test and Spearman's rho) were used for the statistical treatment of the data.

Dilutions of the tissue extracts of the tumours displaced the CRBP standard in a similar way as

Correspondence: G. Fex.
Received 11 October 1985; and in revised form 3 February 1986.

© The Macmillan Press Ltd., 1986

**Table I** Characteristics of the patient material (14 male and 27 female patients aged 47–85 years). Two cases with benign tumours are included in the table.

| Degree of differentiation | *Localization* | | | | *Stage according to Dukes* | | |
|---|---|---|---|---|---|---|---|
| | *Ascendens* | *Trasversum* | *Sigmoideum* | *Rectum* | *A* | *B* | *C* |
| High | 2 | — | 2 | 4 | 1 | 5 | 2 |
| Intermediate | 5 | 3 | 5 | 10 | 5 | 9 | 9 |
| Low | 5 | — | 4 | 1 | — | 5 | 5 |

**Figure 1** Radioimmunoassay standard curve for human liver CRBP (●) and displacement of 125-I-CRBP by dilutions of colon cancer extracts (△). The X-axis gives the CRBP concentration in the respective standards. The Y-axis gives the percent antibody-bound 125-I-radioactivity expressed as percent of the zero standard. With the antibody dilution used for the assay, 50–65% of the tracer was bound in the zero standard.

**Figure 2** Scatter diagram of CRBP concentration ($\mu g\, g^{-1}$ tissue protein) samples of A: whole normal colon (9 males, 16 females), B: colon mucosa+submucosa (5 males, 13 females), C: colon muscularis+serosa (5 males, 13 females) and D: colon tumour (13 males, 28 females). The values (median and range) in males and females for whole normal colon were 33, 19–184 (males); 51, 22–88 (females) and for colon tumours 44, 7–126 (males); 46, 12–791 (females). The median is indicated with a horizontal line.

purified CRBP (Figure 1) indicating that an antigenically similar CRBP was present in the tumour tissue extract. Similar deplacement curves were obtained with extracts from normal colonic tissue.

The CRBP values for whole normal colon (Figure 2) were, with one exception, scattered in the interval 18–82 $\mu g\, g^{-1}$ tissue protein. The tumour CRBP concentrations showed a much larger variation with values both below and above the range for whole normal colon. Two benign tumours, one lipoma and one adenoma contained 34 and 28 $\mu g$ CRBP $g^{-1}$ tissue protein. As tested with the Wilcoxon–Mann–Whitney test the CRBP concentration in the tumour was not significantly different from that of whole normal colon or colonic mucosa from the same patient (Figure 2). There was no sex difference in CRBP concentration either in whole normal colon or in colon tumours and there was no difference in CRBP concentration in samples from whole normal colon or colon tumours from different parts of the colon. However, the CRBP concentration in the tumour was significantly higher than in the muscularis of normal colon ($P<0.05$, concentration ratio (median and range): 1.46; 0.39–3.67; $N=18$). The CRBP concentration in normal mucosa was also significantly higher than in normal muscularis of the same patient ($P<0.05$, concentration ratio (median and range): 1.57; 0.47–2.69; $N=18$). Thus, the mucosal cells are richer in CRBP than the other cells of the colon wall.

The absolute values for the CRBP concentration in whole normal colon obtained in this study were similar to the figures obtained previously using necropsy material (Fex & Johannesson, 1984). Thus, results obtained with necropsy material are close to the values obtained with fresh tissue which indicates that in the colon, CRBP is relatively insensitive to proteolysis post mortem.

The concentration of CRBP in the tumour correlated significantly to the concentration in whole normal colon (rho = 0.46; $P < 0.05$; $N = 23$). Whether this means that tumours with high CRBP concentration arise in areas of normal colon with high CRBP concentration or that tumours with high CRBP concentration in some way increase the CRBP concentration of adjacent normal colon is unknown.

The CRBP concentration in both whole normal colon and in the tumours varied over a wide range (Figure 2). The reason for this wide variation is unknown. Among possible contributing causes is heterogeneity within the tumour. There may also be subgroups of tumours which are richer in CRBP than others but which cannot be identified as such at present. We have carefully gone through the four cases which had very high ($> 100\,\mu g\,g^{-1}$) CRBP concentration in whole normal colon and/or colon tumour, without being able to find anything unique about them. To 'correct' the tumour CRBP values for the possible influence of differences in the CRBP concentration of the surrounding normal colon, individual ratios of tumour CRBP concentration and CRBP concentration of whole normal colon, mucosa and muscularis were calculated.

The CRBP concentration ratio tumour/whole normal colon showed significant statistical correlation with the degree of differentiation of the tumour (rho = 0.54; $P < 0.05$; $N = 23$). Thus, in highly differentiated tumours the difference in CRBP concentration between whole normal colon and tumour tends to be higher than in cases with less well differentiated tumours. Whether this means that high CRBP concentration is related to the well differentiated state or that a normal colon with low CRBP concentration more often gives rise to highly differentiated tumours is presently unknown. The demonstration of increased concentration in differentiated as compared with undifferentiated F9 cells (Eriksson, 1984) lends some support to the former explanation.

The CRBP concentration ratio mucosa/mucularis showed significant negative correlation to patient age (rho = −0.58; $P < 0.05$; $N = 18$) suggesting that the CRBP concentration in these two layers becomes more similar with advancing age.

It is evident (Figure 2) that our results from a larger patient series are in contrast to the findings of Palan *et al.* (1980) since we do not find significantly increased CRBP concentration in colon tumours compared to whole normal colon from the same patient and no sex difference either in whole normal colon or in colon tumours. Our data are also in contrast to the findings in DMH-induced rat colon adenocarcinoma (Ong *et al.*, 1978).

Though a direct comparison of absolute values is difficult to make, due to methodological differences, it can be approximately calculated that the results from normal human colon and colon cancer reported by Palan *et al.* (1980) are about one order of magnitude lower than our figures while the figures of Ong *et al.* (1978) for normal rat colon and colon cancer are more comparable with ours.

The reason for the discrepancy between our data and those of Palan *et al.* (1980) is probably the limited number of patients in their study and the low specificity and sensitivity of their assay. Our data are also at variance with the studies on DMH-induced rat colon adenocarcinoma (Ong *et al.*, 1978). The reason for this may be that, apart from methodological and species differences, the DMA-induced cancers may constitute a more homogeneous tumour group than the tumours in our patients. It may well be that, in man also, there are subgroups of tumours which have higher or lower CRBP concentration than adjacent normal mucosa but, at present, there are no ways of identifying them as such.

Several epidemiological studies have suggested a relationship between vitamin A intake and cancer incidence and between the levels of serum retinol and later cancer development (Willett & MacMahon, 1984). The strongest relationship has been found with lung cancer. Studies aimed at finding such a relationship between colon cancer and vitamin A intake (Modan *et al.*, 1981) or low serum retinol (Nomura *et al.*, 1985) have, however, been negative. Whether this is in any way related to the lack of difference in CRBP concentration between normal colon and colon cancers is unknown.

Supported by grants from the Swedish Medical Research Council (03X-03364), the Medical Faculty, University of Lund, the Albert Påhlsson Foundation, the Malmö General Hospital Cancer Foundation, the Magnus Bergwall Foundation and the John and Augusta Persson Foundation. The skilled technical assistance of Mrs Gunvor Johannesson is gratefully acknowledged.

### References

ACKERMAN, L.V. & DEL REGATO, J.A. (1947). Cancer. Diagnosis, treatment and prognosis. First Edition, C.V. Mosby, St. Louis.

BOYD, D., COPESTAKE, P., CHRISHOLM, G.D. & HABIB, F.K. (1985). A comparison of retinol binding in human hyperplastic and malignant prostate. *Br. J. Cancer*, **51**, 903.

BRADFORD, M.M. (1976). A rapid and sensitive method for the quantitation of microgram quantities of protein using the principle of dye-binding. *Anal. Biochem.*, **72**, 248.

CHYTIL, F. & ONG, D. (1984). Cellular retinoid-binding proteins. In *The Retinoids*, Sporn & 2 others (eds) p. 90. Academic Press.

ERIKSSON, U. (1984). Functional studies of intracellular retinoid-binding proteins. Thesis Uppsala University, Uppsala, Sweden.

FEX, G., LINELL, F. & LJUNGBERG, O. (1985). Cellular retinol-binding protein (CRBP) in normal and neoplastic mammary gland. *Breast Cancer Research and Treatment*, **6**, 131.

FEX, G. & JOHANNESSON, G. (1984). Radioimmunological determination of cellular retinol-binding protein in human tissue extracts. *Cancer Res.*, **44**, 3029.

IKEZAKI, K., UEDA, H., KOYANOGI, T. & 5 others (1985). Cellular vitamin A-binding protein in liver tumors and adjacent tissues. *Cancer*, **55**, 2405.

KÜNG, W.M., GEYER, E., EPPENBERGER, U. & HUBER, P. (1980). Quantitative estimation of cellular retinoic acid-binding protein activity in normal dysplastic and neoplastic human breast tissue. *Cancer Res.*, **40**, 4265.

MODAN, B., CHUCKLE, H. & LUBIN, F. (1981). A note on the role of dietary retinol and carotene in human gastrointestinal cancer. *Int. J. Cancer*, **28**, 421.

MOON, R.C., McCORMICK, D.L. & MEHTA, R.G. (1983). Inhibition of carcinogenesis by retinoids. *Cancer Res.* (Suppl.), **43**, 2469s.

NOMURA, A.M.Y., STEMMERMAN, G.N., HEILBRUN, L.K., SAKELD, R.M. & VUILLEUMIER, J.P. (1985). Serum vitamin levels and the risk of cancer of specific sites in men of Japanese ancestry in Hawaii. *Cancer Res.*, **45**, 1409.

ONG, D.E., CROW, J.A. & CHYTIL, F. (1982). Radioimmunochemical determination of cellular retinol – and cellular retinoic acid-binding proteins in cytosols of rat tissues. *J. Biol. Chem.*, **257**, 13385.

ONG, D.E., GOODWIN, J.W., JESSE, R.H. & GRIFFIN, A.C. (1982). Presence of cellular retinol and retinoic acid-binding proteins in epidermoid carcinoma of the oral cavity and oropharynx. *Cancer*, **49**, 1409.

ONG, D.E., MARKERT, C. & CHIN, J.-F. (1978). Cellular binding proteins for vitamin A in colorectal adenocarcinoma of rat. *Cancer Res.*, **38**, 4422.

PALAN, P.R., DUTTAGUPTA, C. & ROMNEY, S.L. (1980). Sex difference in cellular retinol- and retinoic acid-binding proteins in human colon adenocarcinomas. *Cancer Lett.*, **11**, 97.

PALAN, P.R. & ROMNEY, S.L. (1980). Cellular binding proteins for vitamin A in human carcinomas and in normal tissues. *Cancer Res.*, **40**, 4221.

WILLETT, W.C. & MacMAHON, B. (1984). Diet and cancer – an overview. *New Engl. J. Med.*, **310**, 633.